# Acute Disseminated Encephalomyelitis Following Chemoradiotherapy in an Adult Patient With Nasopharyngeal Cancer

**DOI:** 10.7759/cureus.14137

**Published:** 2021-03-27

**Authors:** Tamar Esakia, Tamar Antia, Marina Janelidze, Armaz Mariamidze, Mikheil Okujava

**Affiliations:** 1 Oncology and hematology, Acad. F. Todua Medical Center, Tbilisi, GEO; 2 Radiology, Acad. F. Todua Medical Center, Tbilisi, GEO; 3 Neurology, S. Khechinashvili University Hospital, Tbilisi, GEO; 4 Pathology, Acad. F. Todua Medical Center, Tbilisi, GEO

**Keywords:** chemoradiation -induced neurotoxicity, nasopharyngeal cancer, brain metastases, acute disseminated encephalomyelitis, adem, chemoradiation-induced demyelination

## Abstract

Acute disseminated encephalomyelitis (ADEM) is a monophasic demyelinating disorder predominantly affecting children. It typically follows a viral illness or vaccination. We present a case of a 34-year-old white male treated with chemo-radiotherapy for nasopharyngeal cancer who developed ADEM. Prompt initiation of intravenous steroids led to the resolution of symptoms and normalization of the brain imaging. We hypothesized that direct brain tissue damage by chemotherapy and radiation therapy, combined possibly with a viral infection, triggered an immune inflammatory response and subsequent demyelination.

## Introduction

Nasopharyngeal carcinoma (NPC) is uncommon, accounting for 0.7% of all cancers diagnosed worldwide in 2018 [1]. Central nervous system (CNS) metastases are rare in NPC, with only a few cases reported in the literature [2-5].

Acute disseminated encephalomyelitis (ADEM) is an immune-mediated inflammatory demyelinating condition predominantly seen in children, which affects the white matter of the brain and the spinal cord [6,7]. Preceding infectious illness or immunization may play a role in the pathogenesis, although a clear preceding event may be absent in up to a quarter of patients [8].

Prompt diagnosis and initiation of treatment is paramount, as in fulminant cases, significant morbidity and even death may ensue. 

We describe a case of ADEM occurring in a patient with NPC after chemo-radiotherapy (CRT) to increase physician awareness for this treatable neuroinflammatory condition.

## Case presentation

**Figure 1:**
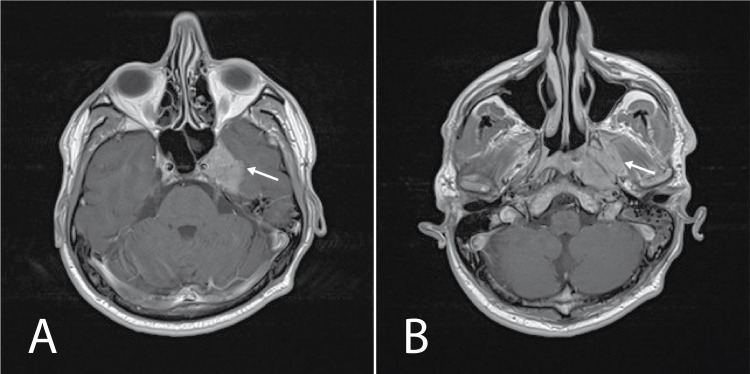
December 2018 initial brain MRI with intravenous contrast. A. a mass with contrast enhancement in the left cavernous sinus, Meckel's cave and medial cranial fossa (arrow) (T1 post contrast sequence); B. a mass with contrast enhancement expanding to pterygoid fossa (arrow) (T1 post contrast sequence).

**Figure 2:**
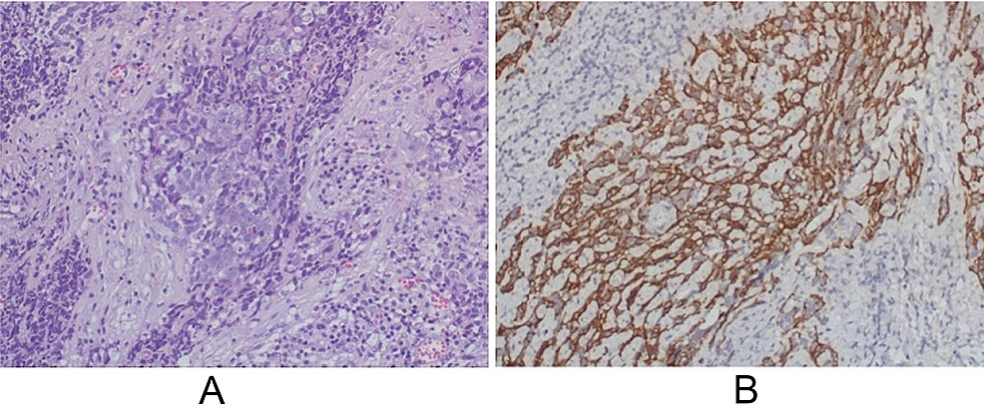
A. H&E (hematoxylin and eosin) stain 20x: poorly differentiated carcinoma; B. CK5 (cytokeratin 5) stain 20x: diffuse expression of cytokeratin five in tumor cells (brown), indicating nondifferentiated nasopharyngeal carcinoma.

A previously healthy 34-year-old white man presented to his primary care physician with a hearing loss on the left side. At the time of presentation, magnetic resonance imaging (MRI) brain with intravenous contrast (IVC) showed extra-axial neoplasm in the left medial cranial fossa extending to the left cavernous sinus and to the pterygoid fossa and the left-sided otomastoiditis and no signs of demyelination (Figure 1). He underwent surgical resection of the tumor and pathology was consistent with non-differentiated NPC, World Health Organization (WHO) type 3 (Figure 2). Staging positron emission tomography-computer tomography (PET-CT) within one month of surgical resection showed one fluorodeoxyglucose (FDG)-avid metastasis in the right lung, and repeat MRI showed brain with IVC showed a residual tumor in cavernous sinus and neck lymphadenopathy.

The patient received four cycles of cytotoxic chemotherapy with cisplatin and 5-flourouracil (5FU). Restaging PET-CT showed stable disease and he was subsequently treated with concurrent chemotherapy/radiation therapy (RT) with cisplatin 40 mg/m^2^ weekly and RT to the pharynx and the left-sided neck lymph nodes with the total dose of 66 Gy given in 33 fractions, and a total of 54 Gy to regional lymph nodes with a daily dose of 2 Gy in 27 fractions.

Two months after completing CRT, he presented with headaches and impaired coordination. He had reported a viral illness two weeks prior with fever (38°C) and chills. He denied vision or hearing loss. A complete review of systems was otherwise negative. His physical exam unremarkable, except for truncal ataxia and horizontal nystagmus. Cranial nerves 2-12 were normal.

**Figure 3:**
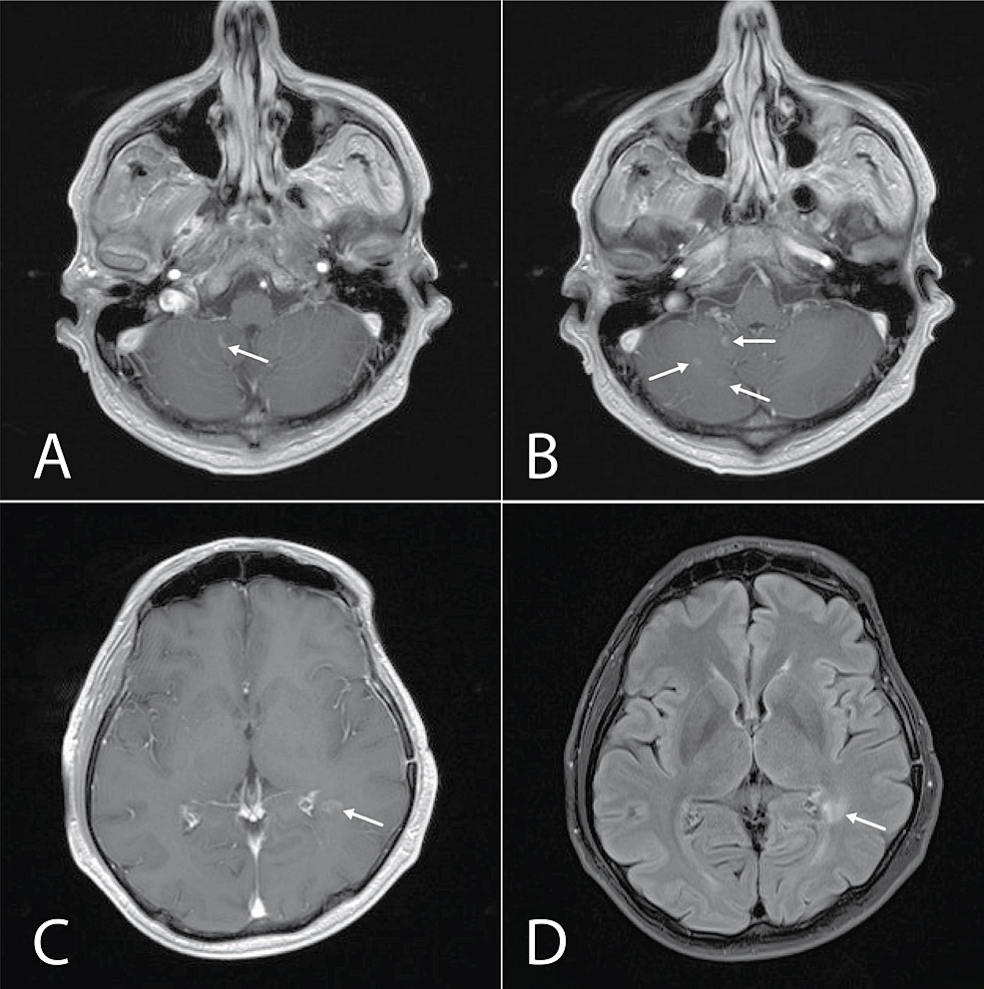
Brain MRI with IVC. A. hyperintense open ring-type non-homogeneous contrast enhancement of cerebellar lesion (arrow) (T1 post-contrast sequence); B. hyperintense non-homogeneous contrast enhancement of cerebellar lesions (arrows) (T1 post-contrast sequence); C. hyperintense open ring-type non-homogeneous contrast enhancement of lesion in left periventricular white matter (arrow) (T1 post-contrast sequence); D. left periventricular lesion without perifocal edema (arrow) (T2/FLAIR sequence). IVC: intravenous contrast; FLAIR: fluid-attenuated inversion recovery

Brain MRI with IVC was performed revealing multiple hyperintense foci in the periventricular white matter and cerebellum. The overall picture was concerning for a demyelinating process. No new mass lesions at the site of initial tumor resection were noted (Figure 3).

**Figure 4:**
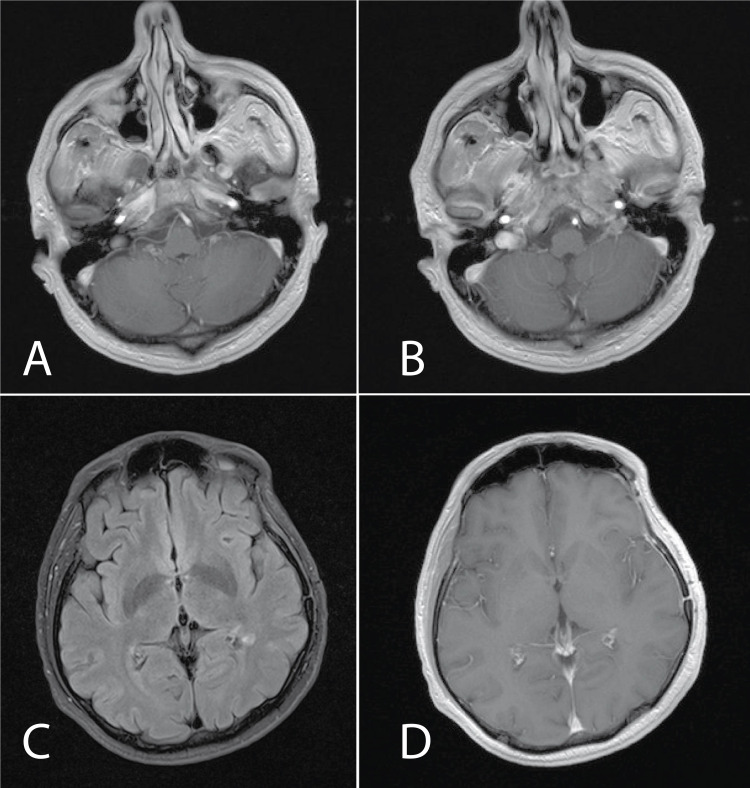
Brain MRI with IVC after treatment with steroids. A. complete resolution of lesion with open ring-type non-homogeneous contrast enhancement in cerebellum (T1 post-contrast sequence); B. complete resolution of hyperintense lesions in cerebellum (T1 post-contrast sequence); C. resolution of left periventricular lesion (T2/FLAIR sequence); D. resolution of left periventricular lesion (T1 post-contrast sequence). IVC: intravenous contrast; FLAIR: fluid-attenuated inversion recovery

A broad differential diagnosis was considered, including brain metastases, but due to the rarity of brain metastasis in NPC and imaging findings argued against metastatic disease. ADEM was diagnosed after consulting with a multidisciplinary team, including a neurologist. The patient declined lumbar puncture for cerebrospinal fluid analysis. Treatment with intravenous dexamethasone 20 mg daily was initiated with a transition to oral formulation and tapering dose resulting in resolution of all symptoms within three weeks. Repeat MRI brain one month later showed complete resolution of the hyperintense foci in the cerebellum (Figure 4). Brain MRI two years later did not reveal any signs of recurrence of ADEM.

## Discussion

Central nervous system (CNS) demyelinating disorders can be primary, such as multiple sclerosis (MS), and secondary (such as infectious, inflammatory, toxic, etc) [9]. Establishing a proper diagnosis through considering differential diagnoses in the clinical context is of particular urgency in patients with cancer, as it affects time-sensitive treatment decisions.

Chemotherapy and radiation therapy can induce neurotoxicity in cancer patients by affecting neural precursor cells, mainly of oligodendrocyte lineage, affecting axonal myelination [10,11]. Chemotherapy depletes oligodendrocyte lineage cells and causes a persistent tri-glial dysregulation by microglial activation and induction of a chronic inflammatory condition, which disrupts the gliogenic microenvironment and glial homeostasis [10]. Activated microglia blocks the proliferation and dysregulates the differentiation of oligodendrocyte precursor cells (OPCs) leading to demyelination. The activation of reactive astrocytes leads to oligodendrocyte death and increases neurotoxicity [10]. The most common chemotherapeutic agents that might cause CNS toxicity manifested as encephalopathy of various severities include methotrexate, vincristine, ifosfamide, cyclosporine, fludarabine, cytarabine, 5-fluorouracil, cisplatin, and the interferons (alpha > beta) [12].

Radiotherapy can cause necrosis of white matter tracts, axonal degeneration, and vascular injury [13]. Radiation-induced demyelination could be the result of the enhanced radiosensitivity of OPCs [11,14]. In addition to this, radiation-induced damage to the microvessels promotes CNS influx of inflammatory cells, causing a pro-inflammatory condition and persistent demyelination [13].

Acute disseminated encephalomyelitis (ADEM) is an immune-mediated inflammatory demyelinating condition that can follow a viral illness. Diagnosis should be considered in patients with cancer who develop typical imaging findings and clinical manifestations. It is considered a monophasic disease, and most commonly affects children, but may occur in adults [15]. Symptoms may be vague and include headache, fever, ataxia, and various sensory and motor symptoms (such as paresthesias and focal motor impairment) [16].

The diagnosis of ADEM is based on clinical and radiological features [6]. MRI is the imaging of choice in ADEM. Hyperintense white matter brain lesions on T2-weighted and FLAIR sequences are characteristic findings [17]. CSF analyses can provide useful information for diagnosis. Pleocytosis and/or increased protein concentration is found in the majority of patients with ADEM [18]. CSF analysis may help rule out MS, though oligoclonal bands can also be present in CSF of patients with ADEM [19]. 

The disease course varies and can be self-limited. High-dose corticosteroids are the mainstay of treatment, with reported full recovery in 50-80% of patients. Typically treatment is initiated with high dose intravenous steroids for the first three to five days and transitioned to oral steroids for three to six weeks in a tapering manner to prevent relapse. Other modalities, such as intravenous immunoglobulin and plasma exchange have also been used successfully if there is no improvement with high-dose steroid use [15, 20].

Our case presented a diagnostic dilemma. Brain metastases were considered, but due to its rarity and no imaging evidence of disease progression elsewhere, the diagnosis seemed less likely. Infectious etiologies were also considered, but the patient was not exhibiting any other signs or symptoms that could support this diagnosis. Chemo-radiation could have contributed to the development of the demyelinating brain lesions in this patient, via the direct toxic effect of CRT or CRT-induced autoimmune phenomenon. ADEM seemed to be the most likely diagnosis, particularly with an antecedent viral infection, and perhaps triggered by prior injury from CRT. Rapid resolution of cerebellar lesions after initiation of steroids without recurrence help support the diagnosis of ADEM.

## Conclusions

ADEM should be considered in patients with cancer, with new neurologic complaints and demyelinating brain lesions on imaging. CRT in association with viral infections could possibly induce ADEM-like demyelination in adults. Further studies are needed to confirm this hypothesis. Proper diagnosis and prompt treatment may lead to quick improvement and avoidance of long term morbidity.
